# Development of a treatment for water contaminated with Cr (VI) using cellulose xanthogenate from E. *crassipes* on a pilot scale

**DOI:** 10.1038/s41598-023-28292-x

**Published:** 2023-02-03

**Authors:** Uriel Fernando Carreño Sayago, Vladimir Ballesteros Ballesteros

**Affiliations:** Fundación Universitaria los Libertadores, Bogotá, Colombia

**Keywords:** Biotechnology, Ecology, Environmental sciences, Engineering

## Abstract

Water care is an imperative duty in industries with effluents loaded with pollutants such as heavy metals, especially chromium (VI), extremely dangerous for humans and the environment. One way of treating water is possible through a continuous system with dry and crushed vegetable biomass of cellulose xanthogenate because it can adsorb heavy metals, especially due to its low production costs. Through continuous systems and with the waste of PET plastics, it is possible to develop a water treatment process adapting this system and biomass. The objective of this research is the development of a treatment for water contaminated with Cr (VI) using cellulose xanthogenate from E. *crassipes* on a pilot scale. Where a mass balance conducted to determine the adsorption capacity of this heavy metal, corroborating it through the Thomas model. The treatment process eliminated around 95% of Cr (VI) present in the water, in addition, biomass reuse cycles carried out, which maintained a considerable adsorption capacity in all the cycles conducted through EDTA reagent.

## Introduction

The world needs a new way of taking care of water due to the incessant growth of cities and industries that pollute water resources. One form of care is innovative treatments that are efficient and cheap. Cellulose biomass is ideal for treating water contaminated with heavy metals due to its ability to chemisorb these contaminants^1^.

Chemical precipitation is the most applied, for the removal of heavy metals. However, this method generates substantial amounts of highly contaminated sludge, and its implementation and operation are very expensive^2^.

Therefore, advanced methods with redefined approaches need in pursuit of sustainable development of industrial expansion. Chemical adsorption is a process that uses materials of biological origin as adsorbents to remove distinct types of contaminants, especially toxic metals^3^.

This technology stands out for being simple, ecological, economical and at the same time flexible to scale, that is, for the continuous treatment of substantial amounts of polluted effluents with low concentrations of metals^1,4^

In different investigations on alternative treatment systems, it was concluding that cellulose has a relevant capacity when it comes to removing heavy metals present in the water due to its power to exchange these contaminants for hydroxyl groups^5–7^^.^ The dried and crushed cellulose residues of E. *crassipes* are an alternative because they have around 70% cellulose, and it is a somewhat expensive residue for final disposal^1,4^^.^ The aquatic plant, E. *crassipes*, expands on the surface of water bodies, creating a layer that limits the transfer of oxygen into the water, affecting ecosystems^8–12^.

The transformation of cellulose with carbon disulfide (CS_2_) forms cellulose xanthogenate, which is an alkaline biomass charged with ions (OH^−^) that allows easy chemisorption of metal cations from industrial wastewater^13,14^. The used the xanthogenate of cellulose was implemented for eliminated Cr (VI)^15^.

The tests to determine the removal efficiencies of heavy metals through this type of biomass are generally carried out in batches, a condition under which the removal efficiencies, adsorption capacities and adsorption kinetics obtained^16^. It is generally carried out on a laboratory scale where their experiments carried out in small volumes (milliliters), from the work at the laboratory level the necessary data can obtained so that, through an adequate scaling technique, a process can defined at the pilot level and this, in turn, serves to define an industrial plant^17^.

Subsequently, column adsorption tests used to establish the efficiency of the biomass in the removal of the contaminant, in an arrangement like that used in the industry^18^. Obtaining rupture curves in specific operating conditions whose behavior depends on the adsorption kinetics, adsorption capacity and hydraulic factors^19^ and in general these tests carried out on a pilot scale, a pilot plant is a process to small scale. The purpose pursued when designing, building, and operating a pilot plant is to obtain information on a certain physical or chemical process, which allows determining if the process is technically and economically viable, as well as establishing the optimal operating parameters of said process. for later design^20^ The adjustment and calibration of continuous treatment systems could couple based on pollutant loads and flow, simulating performance to design processes, using mathematical models^21,22^. The mass balance has used successfully to predict rupture curves, calibrate the input load, and meet discharge standards, being the main parameter to model and define the designs of treatment systems^23,24^.

The complete treatment of system such as the one with fixed columns also consists of improving the treatment conditions by means of EDTA, since it is a substance used as a chelating agent that can form complexes with a metal that has an octahedral coordination structure^25,26^.

The objective of this research is the development of a treatment for water contaminated with Cr (VI) by means of E. *crassipes* xanthate cellulose on a pilot scale. Determining through FTIR and SEM images the characteristics of this biomass, where a mass balance modeled to determine the adsorption capacity of this heavy metal and subsequent elution with EDTA found to determine the ideal concentration for different reuses.

Until now, in the open literature, there were no continuous pilot-scale treatment systems with cellulose xanthate biomass with these characteristics. Although the E. *crassipes* plant has used to remove Cr (VI) in continuous processes^27,28^, these treatment system design parameters were not established. Therefore, the novelty of this study is the implementation of the mass balance, where an equation obtained to determine design variables in construction.

## Methods and materials

### Use the *crassipes*

The leaves and roots were separated from the rest of the plant, washed with abundant tap water, followed by distilled water, where a characteristic population of about 40 plants, already dead, was collected.

The experimental research in plants, including the collection of plant material, complied with the relevant institutional, national and international guidelines and legislation, as stipulated in Decree Law 2376 of 2013^29^, for experimental projects on the environment.

The leaves and roots were cut into small pieces and dried in an oven at 60 °C for 36 h. The pulverized biomass is sieved through a blade mill to obtain different particle sizes of 0.212 mm. The taxonomic level is (*Eichhornia crassipes*). The collection point is the municipality of Mosquera, outside Bogotá DC, located at coordinates: 4.682995, − 74.256673.

Carried out on February 15, 2021, by the researcher Uriel Fernando Carreño Sayago. The plant samples were identified by the research staff (Universidad Los Libertadores) of the Faculty of Engineering, Bogotá, D.C.

### Obtaining cellulose with carbon disulfide (xanthogenate)

The dried and crushed biomass of E. *crassipes* of 40 g, added to 500 ml of NaOH 10 mg/l, then 150 ml of carbon disulfide CS_2_ added. Two variables of NaOH-transformed cellulose (EC-Na) and complete xanthogenate cellulose (EC-X) used. This procedure is based in^13^.

### Chromium measurement

Chromium (VI) laboratory measurement: 50, 100, 200 and 300 mg/L of Cr (VI). Samples were being taken in the flask at each time interval, analyzing the residual chromium concentration. 20 µm samples were taking and then placed in the centrifuge (KASAI MIKRO 200). In sampling, aliquots of the reaction mixture were being analyzed for residual chromium concentration using a UV84.

### Chromium determination

Using the diphenylcarbazide method (the amount of chromium (VI) residue is estimated. For this purpose, the phosphate buffer solution was prepared by adjusting it to a pH equal to 2 with 90% of the grade of purity (H_3_PO_4_). In an eppendorf tube 200 µl of 0.5% diphenylcarbazide (with 97% the grade of purity) and (W/V acetone with 97 of grade of purity), 900 µl of phosphate buffer and 100 µl of the residual sample were added. A suitable portion is transferred to an absorption cell and the absorbance is measured at 540 nm.

### Spectrophotometer (evolution 300 spectrophotometer) by monitoring changes in absorbance

All procedures for the determination of chromium, for water and substrates, were being carried out using the implementation of APHA (Procedure of the American Public Health Association), for standard tests (standard methods for the examination of water and wastewater).

### Adsorption experiments

Adsorption experiments were being carried out in a 100 ml glass vessel with constant stirring (IKA Ks 4000 shaker) at 20 °C, 250 rpm. Data were being taken every 20 min until 180 min were completing. The sample taken is 20 um. All experiments were being carried out in triplicate, with an average of the final values.

The 1000 (mg/l) Cr (VI) stock solution was prepared with distilled water using potassium dichromate. This stock solution used to prepare the test solutions of 200; 300; 400 and 600 mg/l Cr (VI). The batch adsorption study performed at a temperature of 24 °C. The adsorption capacity was determined by suspending 0.3 g of biomass in 100 ml of Cr (VI) solution for 140 min at 200 rpm.

### Continuous experimentation

Development of treatment systems on a pilot scale using PET plastic containers. The treatment systems built using recycled PET containers (600 and 1000 ml). Capsules built where the dried and crushed biomass of ECx and EC-Na.

Each capsule connected to the next through a serial arrangement, with openings in the lids arranged at the bottom of each capsule to allow the flow of treated water to the next capsule.

The flow guaranteed by dripping on the upper capsule, keeping the system flow rate at 15 ml/min, also keeping the pH constant in the feed flow. The bed density was constant, and under temperature and pressure conditions of 20 °C and 1 bar, respectively, the system had manual flow control.

Evaluating initial concentrations of 600, 400, 300 and 200 mg/l of chromium (VI). All tests were performed in duplicate, calculating the average between the data obtained and with this the percentage of metal removal.

For this type of system, three compartments of 15 g each of the different types of biomasses used. In the process of designing and creating these systems, fundamental design parameters adjusted, creating a system that took advantage of the funnel-shaped effect of the containers.

The dry and crushed biomass passed through a 60 mesh, allowing the passage of particles of 0.212 mm in diameter.

The effluent water collected in containers with a capacity of 400 ml, once this capacity covered, the water sample to analyze take and a clean and dry container arranged to continue the collection. Preparations of Cr (VI). The 1000 (mg/l) Cr (VI) stock solution prepared with distilled water using potassium dichromate. This stock solution used to prepare the test solutions of 100 and 400 mg/l Cr (VI).

### Desorption-adsorption

The experimental elution with EDTA carried out after each one of the treatments, when the biomass already presented saturation. The elution consisted of letting the biomass dry and later it passed through the treatment system under the concentration of:

- 1 g/l of reagent in 300 ml of water.
- 3 g/l of reagent in 300 ml of water.
- 5 g/l of reagent in 300 ml of water.

Once the optimal conditions determined, four sorption/desorption cycles performed in order to investigate the regeneration potential of the biomaterial. Between each cycle, the residue washed in a continuous flow of deionized water for 1 h.

### Model evaluation

Mathematical modeling used to describe the behavior of the advance curves, which helps to understand and extend the system. Three different column adsorption models Yoon–Nelson, Thomas and Bohart fitted. To the data of the breakthrough curves to explain the biosorption process of Cr (VI) by EC-Na and ECx in fixed bed configuration.

The Thomas model used to estimate the maximum adsorption capacity and predict the rupture curves, assuming the kinetics of reversible second-order reactions and the Langmuir isotherm^30,31^.

This equation represented for each of the biomasses since it is one of the most representative in the literature to predict the rupture curves and estimate the adsorption capacity, it used to validate the equation.

The Yoon–Nelson model assumes that the adsorption rate decreases proportionally to the adsorbate removal and adsorbent breakdown curve, without considering information such as adsorbate properties, adsorbent type, and adsorption column specifications^32^^.^

The Bohart equation that describes the relationship between C/Co and t in a continuous system has been widely used to describe and quantify other types of systems. This model assumes that the sorption rate is proportional to the residual capacity of the solid and to the concentration of the retained species and used to describe the initial part of the rupture curve^33^ In the Table 1. Show the different model of the adsorptions.

**Table 1:** Models of the adsorptions.

Model Thomas	$\ln \frac{{{\text{Co}}}}{{\text{C}}} - 1 = \frac{{\text{Kth*q*m}}}{{\text{Q}}} - {\text{Kth*Co*Tb}}$
Model Yoon	$\frac{{\text{C}}}{Co} = \frac{1}{{1 + \exp (Kyn\left( {y - t} \right)}}{\text{qN}} = \frac{{{\text{TbCoQ}}}}{m}$
Model Bohart	${\text{Tb}} = \frac{{{\text{No}}}}{CoU}Z - 1\ln \frac{1}{KbCo}{\text{ln}}\left( {\frac{co}{c} - 1} \right)$

Where *Co* Initial concentration of Cr (VI), *C* Final Cr (VI), *V* volume, *KTh* Thomas constant (ml/mg*min), *q* Adsorption capacity (mg Cr /g biomass), *m* Mass of biomass in column (g), *Q* Flow rate through the column (ml/min), *Tb* Time of rupture (min), *K YN* constant Yoon y Nelson (1/h), *q* γ Capacity (mg/g), *Z* bed height (cm), U is the linear flow rate (cm/min1), *Kb* Bohart constant (1/h).

### TESCAN FE-MEB LYRA3

TESCAN FE-MEB LYRA3 scanning electron and focused ion beam microscope. The SEM has an integrated X-ray energy dispersion spectroscopy microanalysis system, EDS (Energy Dispersive X-ray Spectroscopy). (EDS) is an of the most efficient techniques for the qualitative and quantitative analysis of organic samples and through the SEM microphotographs the samples evaluated in the present investigation observed in detail, the diffraction of an X-ray beam by the atoms of the sample interacts with the X-ray beam, producing regions of diffraction intensity, or peaks, for the diagnosis of each of the elements.

## Result

### Analysis of FTIR

**Figure 1:**
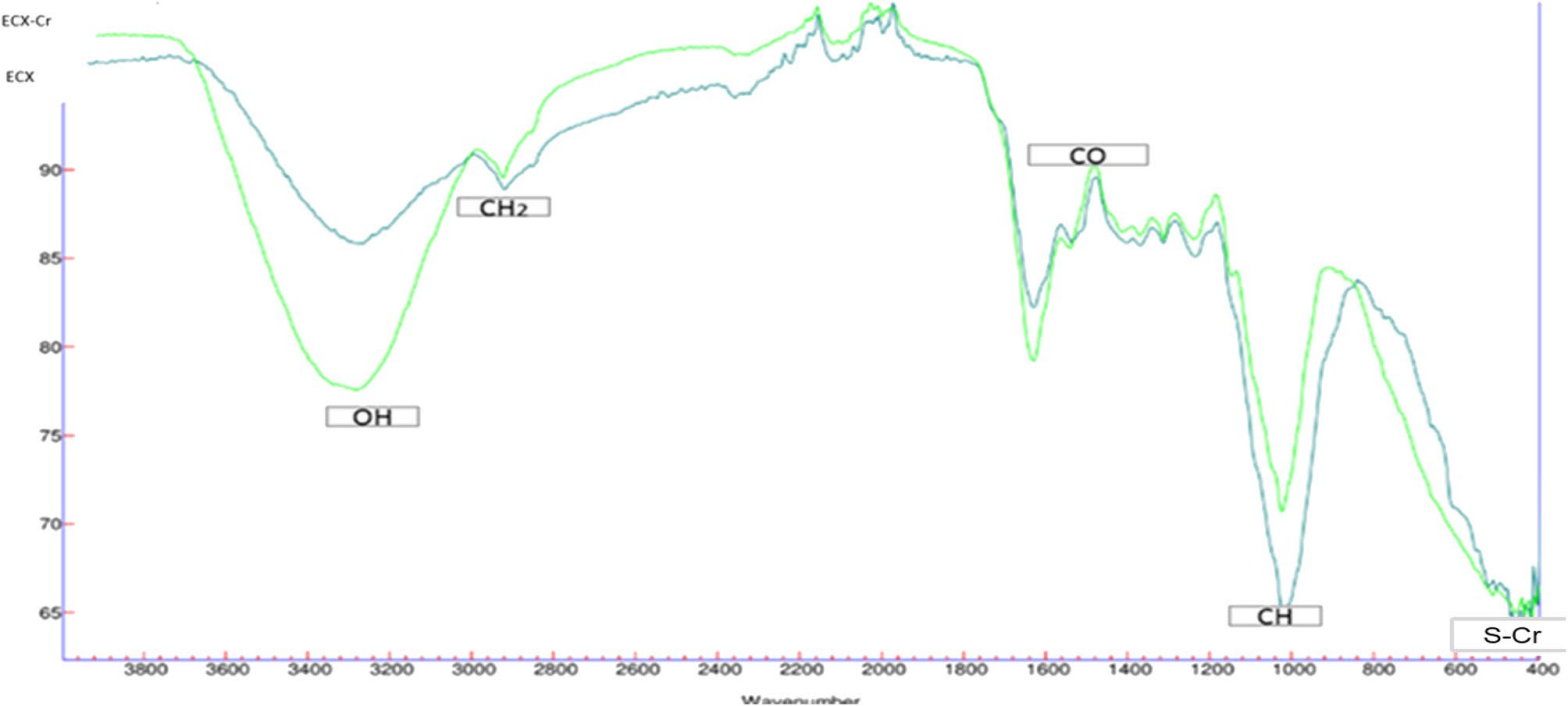
FTIR of ECx before and after of adsorptions of Cr (VI).

Understanding the functional groups involved in the biosorption of toxic metals is essential to elucidate the mechanism of this process. Groups such as carboxylic, hydroxyl and amine are among the main responsible for the absorption of metals by cellulose^34^ In the Fig. 1, show the FTIR of ECx.

According to^13^ the bandwidth at 3000–3600 cm^−1^ corresponds to bonds related to the -OH group. These hydrogen bonds are useful tools for cation exchange with heavy metals. This evidenced in the color spectrum (dark green) that represents an ECx sample with attached Cr (VI) after the adsorption process, where the stretching of the (OH) group lost part of its extension. The change observed in the peak from 3420 cm^−1^ of ECx to 3440 cm^−1^ in ECx-Cr indicates that these groups have a participation in the bond with the Cr (VI) ions. The variation of bands in the peak of the amines after adsorption confirms the participation of these groups in the adsorption process. This result confirmed by the ion exchange evaluation experiment discussed later in section SEM–EDX.

The change in peak 3280, after Cr (VI) adsorption, indicates that EC removed Cr (VI) based on interaction with (OH), part of (OH) lost due to formation of vibrations of ascension O–Cr. Also, after Cr (VI) biosorption on ECx, the peak of the EC-S group is shifted to 590. This can be explained by surface complexation or ion exchange^35^.

In general, comparable results reported in the literature for cellulose in the absorption of other toxic metals, as for other cellulose-derived biosorbentes in the removal of Cr (VI) ions^36^.

One way to corroborate the information presented in the FTIR measurements is through SEM images since with these images it is possible to observe the distribution of the reagents in the ECx biomass treatment and subsequently the Cr (VI) adsorption process.

### SEM–EDX

**Figure 2:**
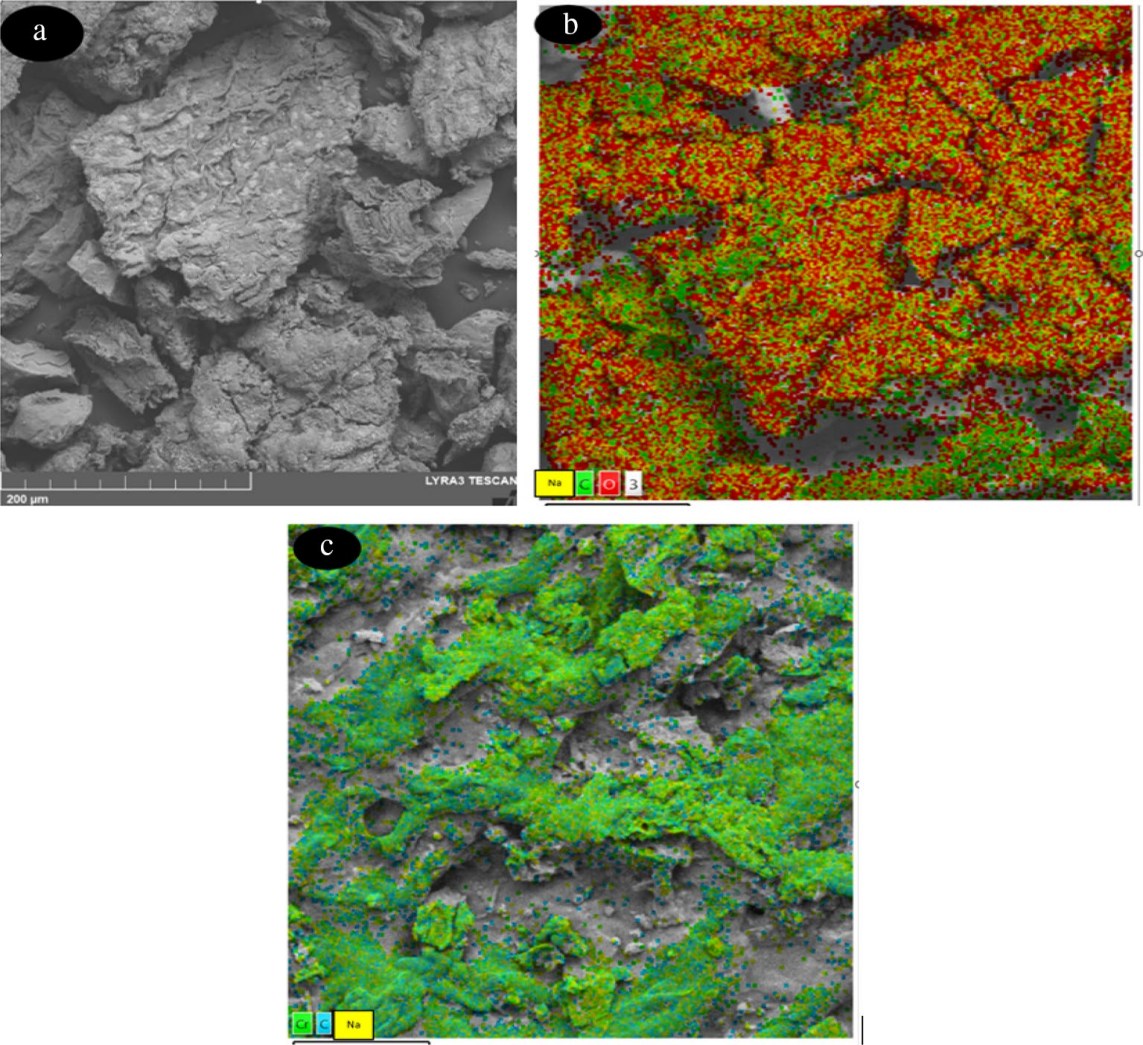
Biomass before (**a**) Cr (VI) adsorption, biomass chemical modifications in (**b**) and shows the distribution of chromium around the whole biomass (**c**).

Figure 2 shows the micrographs obtained for the biomass before (a) the adsorption of Cr (VI), in addition to showing the distribution of the different biomass chemical modifications in (b) and in (c) it shows the distribution of chromium around all biomasses.

From Fig. 2a, it can see that the biomass has a very irregular rough surface, with macropores and cracks. Many of these irregularities may associated with damage caused by the delignification process of E. *crassipes* cellulose with NaOH^14^. In Fig. 2b it is possible to visualize the components of the cellulose xanthogenate, coming from sodium, distributed throughout the biomass, a result like that reported in other studies^35^ The colored dots represent the elements in the samples, green dots represent carbon, red dots represent oxygen, and yellow dots represent the places where sodium lodged.

Table 2 shows that, in addition to carbon and oxygen, the element with the greatest presence in the composition of pure waste is sodium and sulfur from the xanthogenate cellulose transformation process. Table 2 shows the physicochemical characterization of the ECx sample, through EDS.

**Table 2:** Features of sample of ECx.

Element	Weight	Percentage %
Carbon	40.64	44.67
Oxygen	41.15	41.94
Sodium	6.13	6.37
sulfur	5.1	7.3

Cellulose xanthogenate, is one of the cellulose transformations to improve the adsorption performance of heavy metals, this compound produced from dry and ground biomass, mixing with sodium hydroxide (NaOH) to remove lignin, creating alkaline biomass, then disulfide (CS_2_) added^13,14^. (CS_2_) reacts with hydratable hydroxycellulose, forming C-SNa complexes; these are responsible for the cation exchange with heavy metals. Metal ions enter the interior of E. *crassipes* with (CS_2_), exchanging with Na^36,37^.

**Figure 3:**
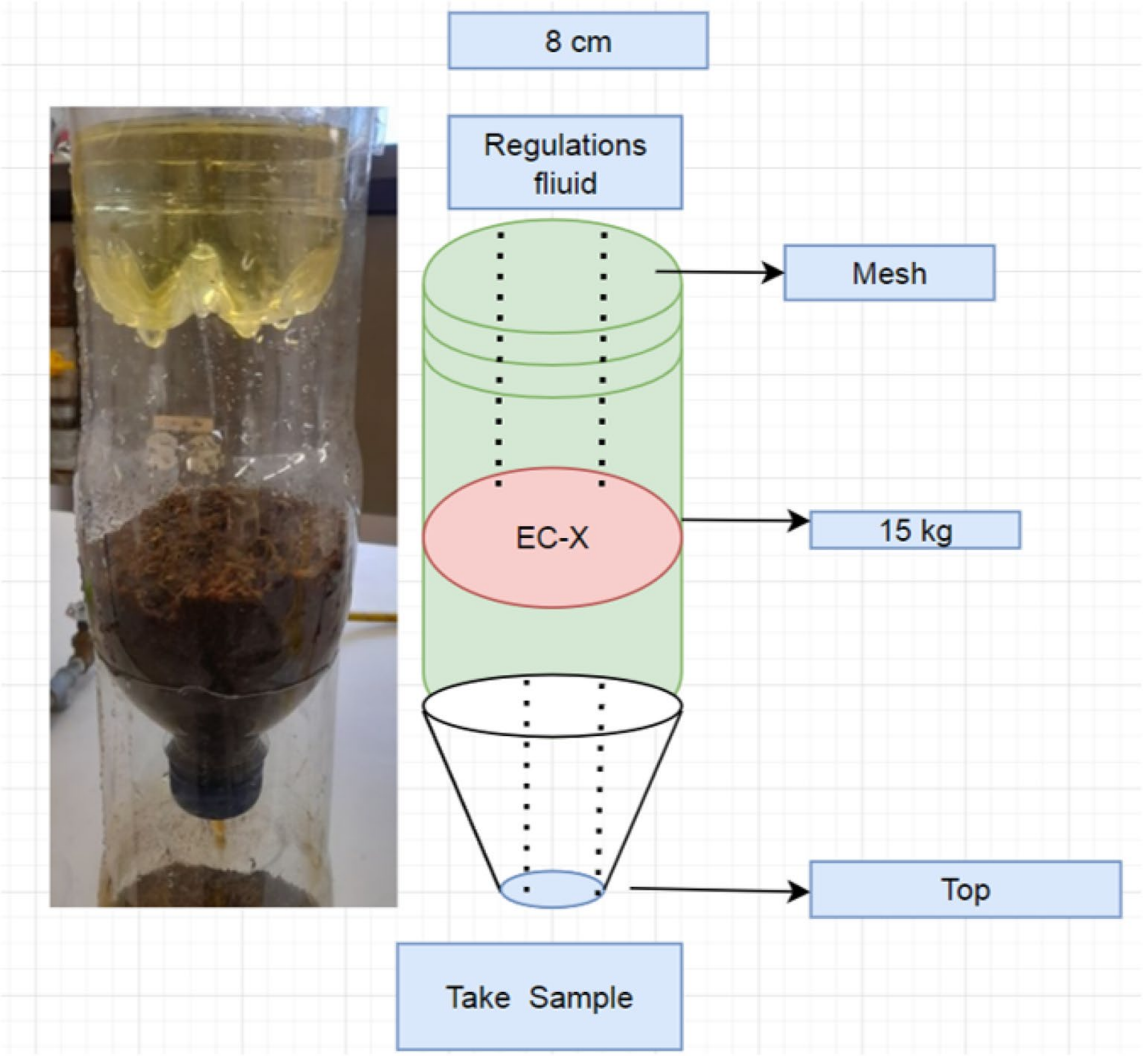
Prototype.

The SEM morphology of ECx and coupled with the high content of sulfides (7.3%) determined by the spectrum in Table 2, it further confirms that xanthate groups are successfully grafted onto the biomass of E. *crassipes,* and Fig. 3 represents this information based on^13,36–38^.

Exchange biochemistry is usually identified as the main mechanism for the adsorption of metals in cellulose and its derivatives^35^ and through the evaluation of EDS this process could verify. Similar observations were made by^36^ where the adhesion of Cr (VI) in this biomass was observed. Also, in xanthogenate cellulose processes, the adhesion of Pb (II) to this type of biomass verified, concluding that this cellulose is important in the removal of heavy metals from water^13^.

The SEM morphology of ECx with Cr (VI) coupled with the high content of sulfides determined by the spectrum in Table 3, was the determinate for the chemisorption’s of Cr (VI). The mechanism of Cr (VI) sorption by cellulose xanthate is:

$$\left[ {{4}\left( {{\text{C}}_{{6}} {\text{H}}_{{{12}}} {\text{O}}_{{6}} } \right)} \right]*{\text{2CS}}_{{2}} {\text{Na }} + {\text{ Cr}}_{{2}} {\text{O}}_{7}^{ - 2} \to \left\{ {\left[ {{4}\left( {{\text{C}}_{{6}} {\text{H}}_{{5}} {\text{O}}_{{6}} } \right)} \right] \, *{\text{2CS}}_{{2}} } \right\}*{\mathbf{Cr}}_{{\mathbf{2}}} + {\text{Na}} + {\text{7H}}_{{2}} {\text{O}}$$

where [4(C_6_H_12_O_6_)] *2CS_2_Na represents the xanthogenate biomass, and Cr_2_O7^–2^ represents the Cr (VI), that 4 parts of glucose xanthate react with the dichromate. In the Tables 3 and 4, the relationship between cellulose xanthogenate and Cr (VI), with related weights of 10.4 for Cr (VI).

**Table 3:** Features of sample of ECx with Cr (VI).

Element	Weigt	%
Carbon	42.6	44.67
Oxygen	30.3	31.94
Chromium	7.13	10.4
Sulfur	4.2	8.2

**Table 4:** Researcher of process of the desorption.

Reference	Biomass	Contaminate Treat	Elution	Capacity (mg/g)	Capacity (mg/g) with the Eq. (6)
Present article	Cellulose xanthogenate (ECx)	Cr (VI)	EDTA	16	51
Present article	Cellulose Alkaline (EC-Na)	Cr (VI)	EDTA	11	32
^39^	*Phanera vahlii*	Cr (VI)	NaOH	30	62
^40^	*A. barbadensis Miller*	Ni (II)	HCl	14	20
^41^	Brown algae	Al (III)	CaCl_2_	12	20
			EDTA		30
			HNO_3_		35
			HCl		35
^42^	Green synthesized nanocrystalline chlorapatite	Cr (VI)	NaOH	20	35
^43^	Graphite	Cr (VI)	HNO_3_	20	52
^44^	Biochar alginate	Cr (VI)	HCl	30	62
^45^	*Pine cone shell*	Pb (II)	HCl	22	30
^46^	Brown algae	Cu (II)	CaCl_2_	14	54
^47^	*Cassava*	Cr (VI)	EDTA	14	40

### Mass balance in treatment

Adsorption is the phenomenon through which the removal of Cr (VI) achieved in the treatment systems; this quantified by means of the general balance equation of the treatment system as shown in Fig. 3.

Adsorption is the phenomenon through which the removal of Cr (VI) achieved in treatment systems, this quantified by mass balance. Equation (1) shows the general balance of matter in the treatment system, together with the accumulation, inputs, and outputs of the system and the chemical process of adsorption.

1 $${\text{Acumulation }}\upvarepsilon *\frac{{\partial {\text{Cr}}\left( {{\text{VI}}} \right)}}{{\partial {\text{t}}}} = {\text{In}} \frac{{\partial {\text{Cr}}\left( {{\text{VI}}} \right)_{0} }}{{\partial {\text{t}}}} - {\text{Out}}\frac{{\partial {\text{Cr}}\left( {{\text{VI}}} \right)}}{{\partial {\text{t}}}} - {\text{Adsortion}}\,{\rho b}\frac{{\partial {\text{q}}}}{{\partial {\text{t}}}}$$

Accumulation represents by Eq. (1), where ∂C(VI) is the contaminant input to the treatment system, (ε) is the porosity of the bed, which calculated as the ratio between the density of the bed of treatment and the density of the microparticle of this biomass. This parameter must be above 0.5^48^ achieved using particle diameters less than 0.212 mm, which favors contact between the contaminant and the particle^49^. The contaminant input to the treatment system represents by the design speed and the amount of contaminant that the system could treat. The output in the treatment system represents by the same input speed and the amount of contaminant that comes out. With these equations, the general material balance will be complete, summarized in Eq. (2), where it can see that the accumulation is equal to the input to the system, minus the output, and minus the adsorption.

2 $$\upvarepsilon *\frac{{\partial {\text{Cr}}\left( {{\text{VI}}} \right)}}{{\partial {\text{t}}}} = \frac{{\partial {\text{Cr}} \left( {{\text{VI}}} \right)}}{{\partial {\text{t}}}} - \frac{{\partial {\text{Cr}} \left( {{\text{VI}}} \right)}}{{\partial {\text{t}}}} - \frac{{\text{M}}}{{\text{V}}}*\frac{{\partial {\text{q}}}}{{\partial {\text{t}}}}$$

where V = System volume (ml), ε = Porosity, Co = Initial concentration of Cr (VI) (mg/ml), C = Final concentration Cr (VI) in the treated solution (mg/ml), Q = design flow (ml/min), Tb = Breaking time (Min), M = amount of biomass used (g), q = Adsorption capacity of the biomass used (mg/g).

3 $${\text{V}}*\upvarepsilon *{\text{Co}} = {\text{Q}}*{\text{Tb}}*{\text{Co}} - {\text{Q}}*{\text{Tb}}*{\text{C}} - {\text{M}}*{\text{q}}$$

Depending on the most important parameters when building a treatment system, Eq. (3) could use to model and validate the best form of treatment, for example, the necessary amount of biomass to use to treat a certain amount of contaminant, in the present investigation it used to establish the adsorption capacity in these initial treatment conditions. The remaining Eq. (4) determines the adsorption capacity.

4 $${\text{q}} = \frac{{{\text{QTbCo}}}}{{\text{M}}} - \frac{{{\text{QTbCf}}}}{{\text{M}}} - \frac{{\upvarepsilon {\text{VCo}}}}{{\text{M}}}$$

Adsorption capacity is generally taken through Eq. (5) for both batch and continuous experiments^20,21^But unlike Eqs. (5), (4) takes into account design variables such as flow rate (Q), rupture time (Tb), particle bed porosity ε, and vessel design volume (v).

5 $${\text{q}} = \frac{{{\text{v}}\left( {{\text{Co}} - {\text{C}}} \right)}}{{\text{m }}}$$

where m: Mass used in the treatment, V: Volume, C_o_: Initial concentration, C: Final Concentration, Q: adsorption capacity.

However, unlike Eqs. (5),  (4) considers the design variables such as flow rate (Q), rupture time (Tb), particle bed porosity ε and vessel design volume (v).

When a desorption-elution process is involved for the reuse of biomass, Eq. (4) would be:

6 $${\text{q}}_{{\text{T}}} = \mathop \sum \limits_{j = 1}^{n} \left[ {\frac{{{\text{QTbjCo}}}}{{\text{M}}} - \frac{{{\text{QTbjCj}}}}{{\text{M}}} - \frac{{\upvarepsilon {\text{VCo}}}}{{\text{M}}}} \right]$$

where Q = design flow (ml/min), Tbj = Break time of use number j (Min), Co = Initial concentration of Cr (VI) (mg/ml), C = Final concentration Cr (VI) in the treated solution (mg/ml), V = System volume (ml), ε = Porosity, M = amount of biomass used (g), q_T = Total adsorption capacity of the biomass used (mg/g).

This model (6) is design to determine the adsorption capacity when different elution processes have conducted, it will used to determine the new adsorption capacity and is one of the contributions of the present investigation.

### Result process of adsorptions

**Figure 4:**
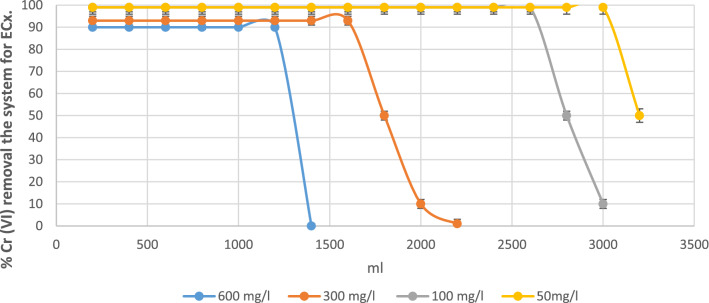
Percentages of Cr (VI) removal the system for ECx.

In Fig. 4 shows the Cr (VI) adsorption process of the system.

Various researchers have extensively studied the influence of factors such as bed height, flow rate and metal inlet concentration on rupture (Tb) curves. For example, the influence and similarity of the initial contaminant concentrations should be reflected as in the case of a tannery, with initial concentrations of 600 mg/l. Figure 4 shows the progress curves obtained for the study of Cr (VI) removal by the studied biomasses, reflecting the percentage of Cr (VI) removal in contrast to the treated volume, which is a very important parameter to time to scale the process.

**Figure 5:**
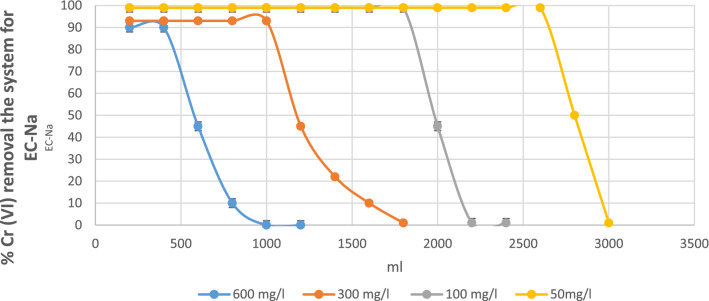
Percentages of Cr (VI) removal the system for EC-Na.

Regarding the effect of the input concentration, it can see in Fig. 5 that the breakpoint had a better performance in all the initial concentrations in the ECx biomass. comparing it with the EC-Na biomass (see Fig. 5), always obtaining breakpoints with more treated volume.

The difference between the rupture curves between ECx and EC-Na indicates that the cellulose xanthate modification scheme should completed, although it can also elucidate that the EC-Na biomass has high yields compared to other biomass studied. for example, in Ref.^34^ investigate the biomass of E. *crassipes* without modifying, having removals below this alkaline cellulose.

### Adsorption capacities

Through Eq. (3), the adsorption capacity of ECx*,* using the initial concentration of 600 mg/l, since it was the maximum concentration used.

**Figure 6:**
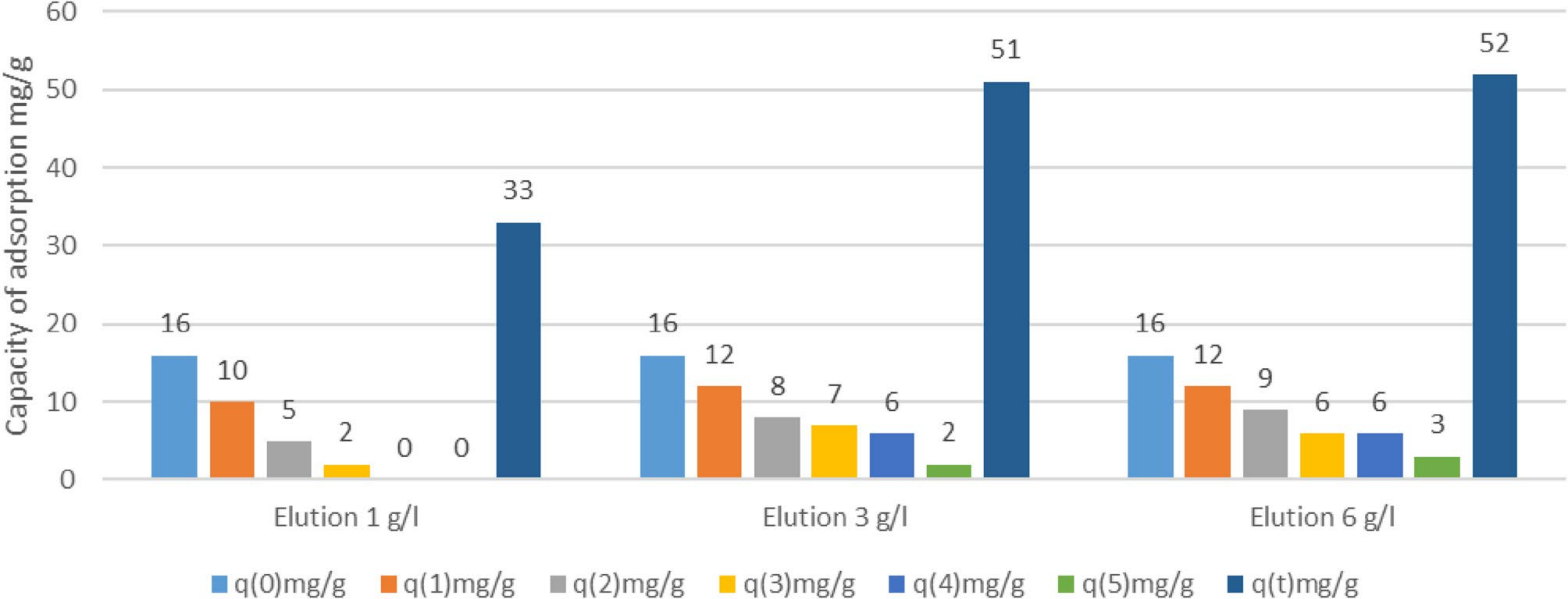
Adsorption capacities in the different adsorption processes in the biomass ECx.

The break point was around 1200 ml according to Fig. 6 and together with the flow rate of 15 ml/min; the break time obtained in 80 min.

$${\text{q}} = \frac{{80{*}15{*}0.6}}{40} - \frac{{80{*}15{*}0.04}}{40} - \frac{{0.66{*}78{*}0.6}}{40}$$

q: Adsorption capacity, Co: 0.6 mg/ml, C: 0.06 mg/ml, M: 40 g, Tb: rupture time 80 min, Q: 15 Flow rate ml/min, ε: 0.66^49^, V: Occupied volume: 70 ml.

A result of 16 mg/g obtained in this continuous study for the biomass ECx. With this same equation it gives the capacity of the biomass EC-Na, with 11 mg/g.

### Desorption-Elution and reuse

Through Eq. (6), the sum of the Cr (VI) adsorption capacities established, after different biomass reuses due to EDTA elution. In the second treatment process, it yielded the following results under concentrations of 6 g/l of EDTA.

$${\text{q}}\left( {\text{T}} \right) = \frac{{60{*}15{*}0.6}}{40} - \frac{{50{*}15{*}0.06}}{40} - \frac{{0.66{*}68{*}0.6}}{40}$$

Co: 0.6 mg/ml, C: 0.06 mg/ml, M: 45 g Biomass eluted with EDTA, Tb: rupture time: 60 min, Q: 15 Flow ml/min, ε: 0.66^49^, V: Occupied volume: 68 ml, q: 10 mg/g.

Five Cr (VI) adsorption cycles performed using ECx and EC-Na cellulose in a continuous system to evaluate the regeneration and reuse potential. Between each biosorption cycle, a desorption cycle performed using three different concentrations of EDTA eluent.

**Figure 7:**
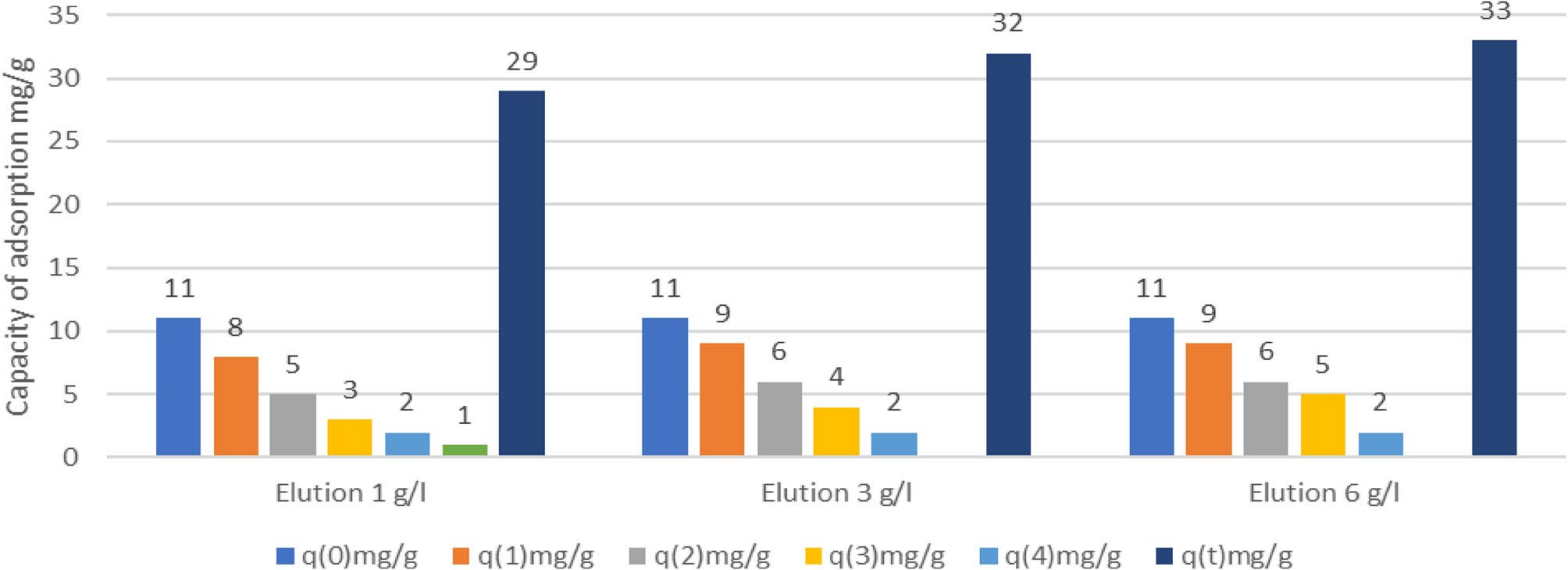
Adsorption capacities in the different adsorption processes in the biomass EC-Na.

According to Figs. 6 and 7, although the adsorption capacity gradually decreases from the first adsorption process, it could consider that it is a satisfactory biomass recycling process and a design parameter for later stages of this treatment system.

In the experiments with concentrations of 6 g/l, five reuse processes obtained, obtaining a final sum of 52 mg/g. In concentrations of 3 g/l of EDTA, final capacities of 51 mg/g obtained lower than concentrations of 6 g/l but with half of this reagent. With concentrations of 1 g/l, final capacities of 33 mg/g obtained.

The desorption processes of the EC-Na biomass with initial capacities of 11 mg/g were also evaluated and through desorption processes with EDTA of 3 g/l this biomass recycled on 5 occasions, reaching 32 mg/l in capacities of adsorption and like the EC-Na biomass, the ideal concentration in the process for desorption processes is 3 g/l, due to the considerable increase in reuse processes and low concentration compared to 6 g/l, which, although higher, does not this value is significant in the absorption capacity.

Through Eq. (6) and with different bibliographic references, representative data obtained to feed this equation, determining the capacities of each of these biomasses together with the new capacities determining the desorption power of the different eluents shown and summarized in Table 4.

For the EDTA eluent and with Eq. (6), satisfactory results evidenced by removing Al (II), reaching almost 150% of its adsorption capacity, corroborating what presented in the present investigation, also the EDTA reagent obtained interesting yields to recycle the cassava biomass increasing up to 40 mg/g. In Ref.^39^ used the biomass of *Phanera vahlii* to remove Cr (VI) obtaining results of 30 mg/g and with NaOH they reached capacities in the reuse process of this biomass up to 62 mg/g, reaching almost double of its total capacity^41^, also used NaOH for desorption processes with green synthesized nanocrystalline chlorapatite biomass, achieving results of 75% more. The eluent HCl is also a good chemical agent to use in desorption processes since it reached more than 100% in the reuse of biochar alginate for Cr (VI) but not so significant with biomass A. *barbadensis Miller* to remove Ni (II) and in^40^ significant results were also obtained to remove Pb (II) with *pine cone* Shell biomass. With the chemical agent HNO_3_, interesting contaminant recycling processes obtained, since more than 100% of the adsorption capacity of the biomasses used in this process used^1,45^.

### Mathematical models of adsorption

In general, the models presented R^2^ greater than 0.95 for the adjustment of all the advance curves, which indicates a good adherence to the data, the model that best describes the behavior of the ECx system was the phenomenological model Thomas, which presented all the R^2^ values above 0.99.

This model could use for the extension of the Cr (VI) ion biosorption system using cellulose xanthogenate, in the literature it is possible to observe that this model often tends to better adapt to the experimental data of the adsorption systems that use cellulose for the absorption of toxic metals^28,30,31^.

With q_t_ values remarkably close to the experimental values of Eq. (4) designed and presented in this investigation, indicating the validity of this equation where it reflects the maximum capacity obtained. Table 5 shows the adsorption constant of the Thomas model (K_t_), which corresponds to the adsorption rate of Cr (VI) in the biomass^49^ This value was 0.048 (ml/mg*min) reflecting the speed with which Cr (VI) is chemisorbed in the biomass of ECx, in the EC-Na cellulose there was a Thomas model speed of 0.039 (ml/ mg*min) evidencing a lower adsorption rate than ECx. In the adsorption of Cr (VI) by rice biomass, the Thomas constant is 0.1 (ml/mg*min)^47,50^ also in the adsorption of Cr (VI) by biomass. Nanocrystalline chlorapatite biomass obtained at the Thomas constant 0.013 (ml/mg*min)^49^.

**Table 5:** Summary of the experiments obtained with material ECx.

Model	Parameters	50 (mg/l)	100 (mg/l)	300 (mg/l)	600 (mg/l)
Thomas	R^2^	**0.99**	**0.99**	**0.98**	**0.97**
	k_t_	0.048	0.044	0.046	0.049
	q_t_	17	16.5	16.3	17.2
Bohart	R^2^	0.88	0.98	0.97	0.9
	k_b_	0.962	0.954	0.974	0.9542
Yoon	R^2^	0.9	0.8	0.94	0.93
	k_y_	0.944	0.933	0.98	0.93

Significance values are in bold.

In the Table 6, it presents summary of the experiments obtained with material EC-Na.

**Table 6:** Summary of the experiments obtained with material EC-Na.

Model	Parameters	50 (mg/l)	100 (mg/l)	300 (mg/l)	600 (mg/l)
Thomas	R^2^	0.97	0.98	0.95	0.95
	K	0.038	0.034	0.036	0.039
	q_t_	7	7	8	9
Bohart	**R**^2^	**0.99**	**0.99**	**0.98**	**0.97**
	k_b_	0.862	0.854	0.874	0.8542
Yoon	R^2^	0.88	0.9	0.91	0.92
	k_y_	0.844	0.833	0.88	0.83

Significance values are in bold.

The Cr (VI) adsorption process in the EC-Na biomass represented through the Bohart equation, since the sorption rate is proportional to the biomass capacity, obtaining an adsorption rate of 0.85(ml/mg*min). Having an alkalized biomass represents this model due to the homogeneity of this adsorbent.

### Mathematical models in desorption processes

The continuous desorption process with its fit to the Thomas model for biomass ECx always shows the fit of this model with significance, because this type of model fits representatively to desorption processes with good performance^32,51^ It can also verify that with values of q_t_ it is close to the experimental values of Eq. (6) designed and presented in this research, indicating the validity of this equation again, where it reflects the maximum capacity obtained.

In the Table 7. Show Summary of the experiments obtained with material ECx in process of desorption’s.

**Table 7:** Summary of the experiments obtained with material ECx in process of desorption’s.

Model	Parameters	Cycles				
		1	2	3	4	5
Thomas	R^2^	0.99	0.99	0.98	0.97	0.99
	k_t_	0.048	0.044	0.036	0.033	0.03
	**q**_**t**_	**12**	**11**	**8**	**6**	**4**
Bohart	R^2^	0.88	0.98	0.97	0.9	0.88
	k_b_	0.962	0.854	0.804	0.77	0.66
Yoon	R^2^	0.9	0.8	0.94	0.93	0.90
	k_y_	0.944	0.88	0.80	0.75	0.7

Significance values are in bold.

In the Table 8 the EC-Na biomass had a different behavior and in its second and third cycle it adjusted to the Yoon model and later to the Bohart model.

**Table 8:** Summary of the experiments obtained with material EC-Na in process of desorption’s.

Model	Parameter	Cycles			
		1	2	3	4
Thomas	R^2^	0.91	0.90	0.85	0.85
	k_t_	0.038	0.034	0.025	0.022
	q_t_	7	7	6	5
Bohart	R^2^	0.99	0.99	0.98	0.97
	k_b_	0.862	0.854	0.77	0.66
Yoon	R^2^	0.88	0.9	0.91	0.92
	k_y_	0.844	0.80	0.77	0.63

This behavior is due to the alkalinization of the biomass and this process makes the biomass a little more unstable. The values of q_t_, although a resemblance evidenced, were not so representative due to the little adjustment that there was with respect to the Thomas model.

## Conclusions

In this work, a treatment system designed and evaluated to remove Cr (VI) through cellulose from E. *crassipes* xanthate. SEM/EDX analysis tests showed that ion exchange mechanisms involved in the removal of Cr (VI) by ECx and EC-Na. The regeneration and reuse studies of sorbents showed that the removal capacity remained satisfactory in the five cycles conducted, being the 3 g/l EDTA solution the best result for the reuse of the evaluated biomasses. The model developed in the present investigation through mass balance validated through the Thomas model since this model was the one that best described the adsorption process. Concluding that cellulose xanthate is a promising alternative biomass for the removal of Cr (VI) ions in a dynamic system. Future studies should carry out on larger scales with a view to industrial application, since this biomass presents, in addition to its good adsorption capacity, several advantages such as low cost, high availability, and promising regeneration/reuse.

## Data Availability

The datasets used and/or analyzed during the current study available from the corresponding author on reasonable request.
